# The influence of a single hemodialysis procedure on human T lymphocytes

**DOI:** 10.1038/s41598-019-41619-x

**Published:** 2019-03-25

**Authors:** Katarzyna A. Lisowska, Małgorzata Pindel, Krzysztof Pietruczuk, Izabella Kuźmiuk-Glembin, Hanna Storoniak, Alicja Dębska-Ślizień, Jacek M. Witkowski

**Affiliations:** 1 Department of Pathophysiology, Faculty of Medicine, Medical University of Gdańsk, Gdańsk, Poland; 2 Department of Nephrology, Transplantology and Internal Medicine, Faculty of Medicine, Medical University of Gdańsk, Gdańsk, Poland

## Abstract

At the moment it is unknown to what extent the impaired function of T lymphocytes in ESRD patients depends on uremia, and to what extent on hemodialysis (HD) procedure. Therefore, the purpose of the study was to evaluate percentages of T lymphocyte subpopulations *ex vivo*, plasma concentrations of IL12p70, TNF, IL-10, IL-6, IL-1β, IL-8 cytokines and selected proliferation parameters of *in vitro* activated T lymphocytes in HD patients before and after single HD procedure using flow cytometry. We demonstrated that the percentage of CD8^+^ cells *ex vivo* was decreased while the CD4^+^/CD8^+^ ratio was increased after HD procedure. Also, there was significant decrease in the percentage of CD8^+^HLA-DR^+^, CD8^+^CD69^+^ and CD8^+^CD95^+^ cells after HD. At the same time, an increase in the percentage of CD4^+^CD95^+^ cells was observed after HD. From all analyzed cytokines, only the concentration of IL-8 was significantly decreased after HD procedure. A single HD session enhanced proliferation capacity of CD4^+^ cells but not CD8^+^ cells *in vitro* by increasing number of cell divisions and percentage of dividing cells. Our results show that a single hemodialysis can have immunomodulatory effect on HD patients and may contribute to the state of immune deficiency observed in patients with ESRD.

## Introduction

Chronic kidney disease (CKD) is characterized by the slow loss of kidney function over a period of years that leads to its fifth stage – end-stage renal disease (ESRD). Patients at this stage require renal replacement therapy, which is usually hemodialysis, to reduce the side effects of kidney disease, especially uremia, fluid volume overload and metabolic acidosis. Progression of CKD also promotes the development of disorders in the immune responses. Infections are a second leading cause of death among hemodialysis (HD) patients, mainly due to the impairment of both innate and acquired immunity^1^. Patients are also characterized by weakened response to vaccinations; especially, the effectiveness of hepatitis B virus (HBV) vaccination was found to be lower in ESRD patients compared to healthy subjects^2^. It is believed that the main cause of immune deficiency is the accumulation of uremic toxins, a process which is associated with the progression of renal disease^3^. Though, it seems that it may also be a result of repeated hemodialysis treatments.

It is believed that synthetic membranes (e.g., polysulphone membrane) are generally characterized by a better biocompatibility compared to cellulosic membranes (e.g. cuprophan membrane, or membranes from modified cellulose, e.g. hemofan membrane), since no change is observed in the number of monocytes or lymphocytes in a patient’s blood after hemodialysis^4^. But some studies show that when blood flows through the dialyzer, the complement system components and immune cells, especially neutrophils and monocytes, are activated, which leads to the release of large amounts of pro-inflammatory cytokines, IL-1, IL-6 and TNF-α^5^. Moreover, an increase of pro-inflammatory IL-6 and TNF-α production by whole blood cells with a simultaneous deficiency of anti-inflammatory IL-10 is also observed *in vitro*^6,7^. Circulating monocytes of ESRD patients present elevated expression of Toll-like receptors (TLR2 and TLR4)^8^, which also supports the thesis of monocytes and macrophages of HD patients spontaneously activated, probably during the procedure. At the same time, ESRD results in depletion and dysfunction of dendritic cells, especially plasmacytoid DCs, which is further aggravated by HD treatment^9^. Disturbances in the acquired response are also observed; CD4^+^ T lymphocytes of HD patients are characterized by a reduced expression of key surface antigens (especially co-stimulatory CD28 and activation markers: CD69 and CD25) and impaired proliferation parameters (including reduced number of cell divisions, longer period of time required by these cells to enter the first (G1) phase of the first cell cycle and decreased percentage of cells able to divide) in response to polyclonal stimulation of the TCR/CD3 complex^10,11^. Since the function of B lymphocytes largely depends on the activation of CD4^+^ cells, their impaired activity can encourage the progression of immune deficiency manifested as a reduced response of HD patients to vaccination against hepatitis B^2^, tetanus^12^ or pneumococcus^13^. Indeed, our latest results show that T cell-dependent activity of B cells is significantly impaired in HD patients^14^. Reasons of these abnormalities are still unclear. Apparently the problem is complicated and includes at least two major ideas: improperly working kidneys leading to uremic state, which has global effect on organism, and direct contact of dialysis membrane with peripheral blood mononuclear cells (PBMCs) leading to abnormal production of pro-inflammatory cytokines and other inflammatory mediators.

Presently, hemodialysis is the most common method of renal replacement therapy in patients with acute and chronic renal failure in Poland. At the moment, it is still unknown to what extent the impaired function of T lymphocytes in ESRD patients can be caused by the repeated hemodialysis sessions. Especially, the effect of a single HD session on T lymphocytes is unclear. Recent studies have shown that after HD session the percentage of CD25^+^ cells along with plasma level of IL-6 increases in diabetic patients^15^, which suggest that even a single hemodialysis procedure can prime an activation of peripheral blood lymphocytes. Therefore, our pilot goal was to evaluate percentages of selected T lymphocyte subpopulations *ex vivo* and plasma concentrations of IL12p70, TNF, IL-10, IL-6, IL-1β, IL-8 cytokines in ESRD patients on maintenance hemodialysis treatment, before and after a single HD session. Among T lymphocyte subpopulations, we chose CD28 antigen because of its co-stimulatory role in antigen presentation and markers of T lymphocyte activation: early – CD69, middle – CD25, CD95 and late – HLA-DR. Additionally, we analyzed proliferation capacity of *in vitro* stimulated T lymphocytes obtained before and after a single HD in order to establish whether single procedure could influence their activity.

## Results

### Comparison of main subpopulations of T lymphocytes and cytokine levels *ex vivo*

**Figure 1:**
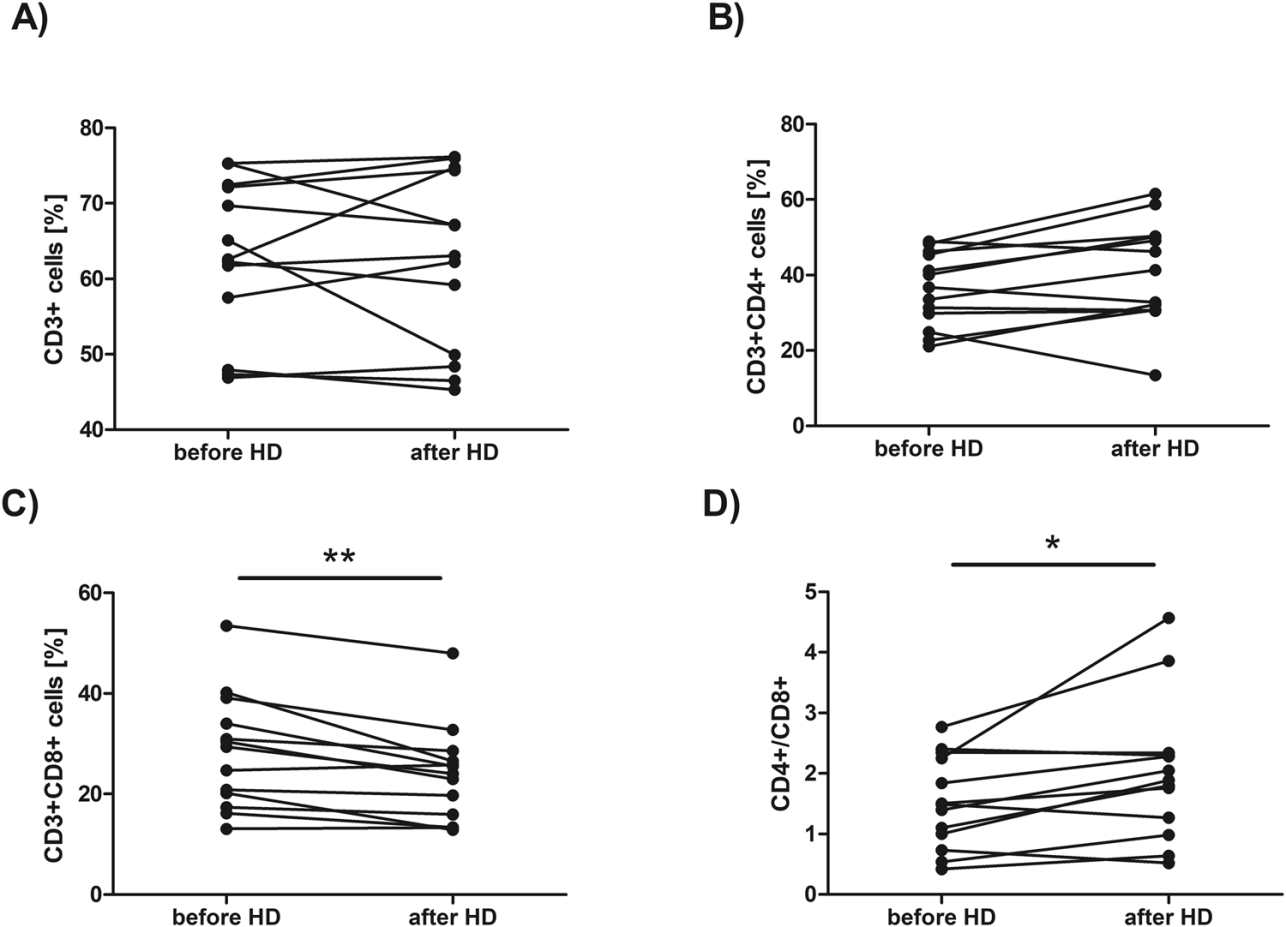
Comparison of the percentage of CD3^+^ (**A**), CD3^+^CD4^+^ (**B**), CD3^+^CD8^+^ (**C**) cells and CD4^+^/CD8^+^ ratio (**D**) in the blood sample taken before and after hemodialysis. Each line shows individual values of cell percentage. Differences statistically significant are marked with *(p < 0,05) or **(p < 0,01), Wilcoxon signed-rank test.

No difference has been found in the percentage of CD3^+^ cells (the median value was 63,58 before HD *vs* 63,04 after HD, Fig. 1A) as well as in the percentage of CD3^+^CD4^+^ cells (the median value was 36,79 before HD *vs* 41,30 after HD, Fig. 1B) *ex vivo* after HD session. At the same time, the percentage of CD3^+^CD8^+^ cells (Fig. 1C) was significantly decreased after HD session (the median value was 29,37 before HD *vs* 24,03 after HD, p = 0,002977, Wilcoxon signed-rank test). The CD4^+^/CD8^+^ ratio (Fig. 1D) was also increased after HD (the median value was 1,49 before HD *vs* 1,89 after HD, p = 0,013104, Wilcoxon signed-rank test) immediately after HD procedure (Fig. 1D). No difference has also been found in the percentage of CD3^+^CD4^−^CD8^−^ cells (the median value was 33,08 before HD *vs* 32,61 after HD).

**Figure 2:**
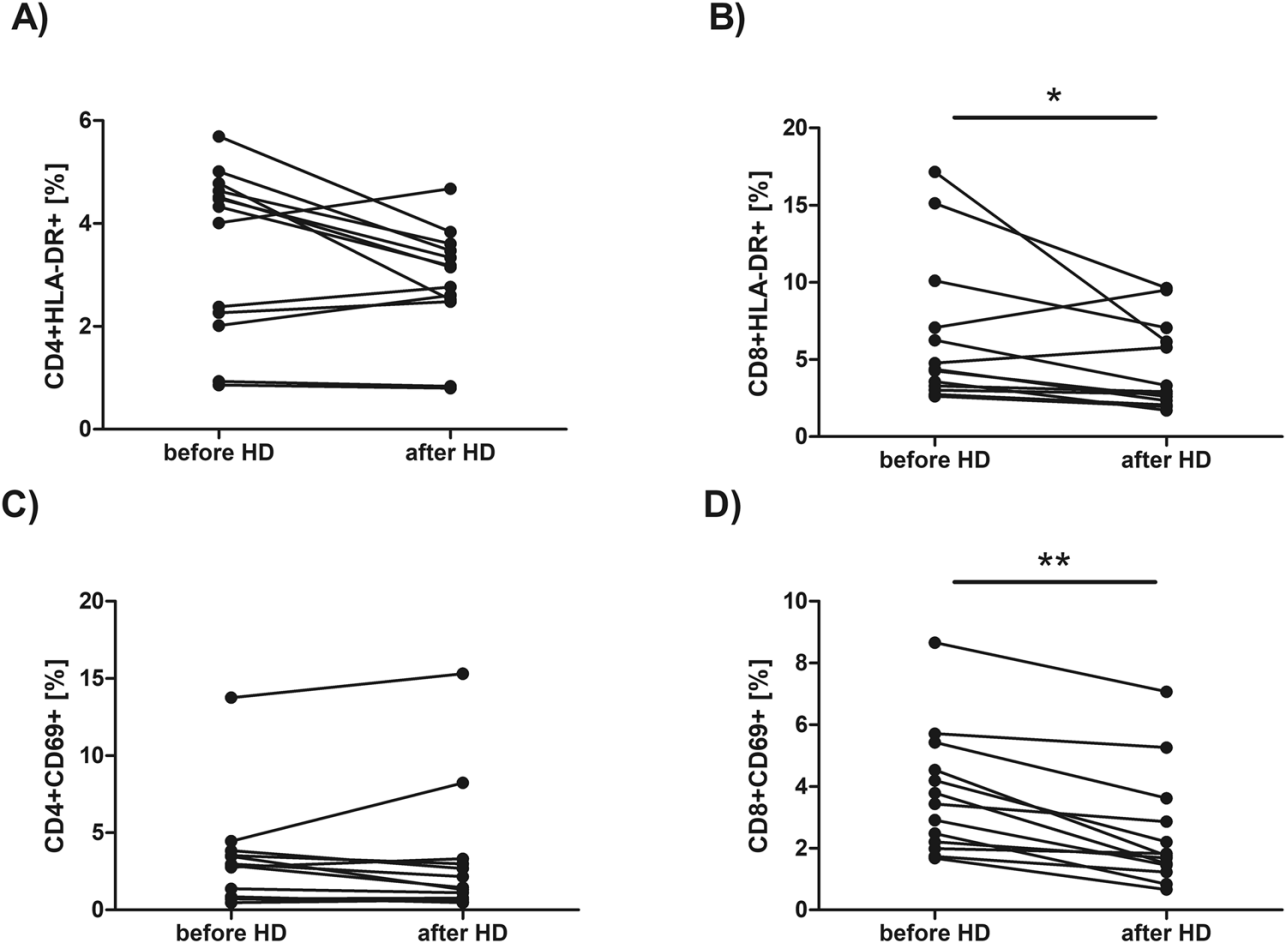
Comparison of the percentage of CD4^+^ and CD8^+^ with the expression of HLA-DR (**A**,**B**) or CD69 (**C**,**D**) in the blood sample taken before and after hemodialysis. Each line shows individual values of cell percentage. Differences statistically significant are marked with *(p < 0,05) or **(p < 0,01), Wilcoxon signed-rank test.

**Figure 3:**
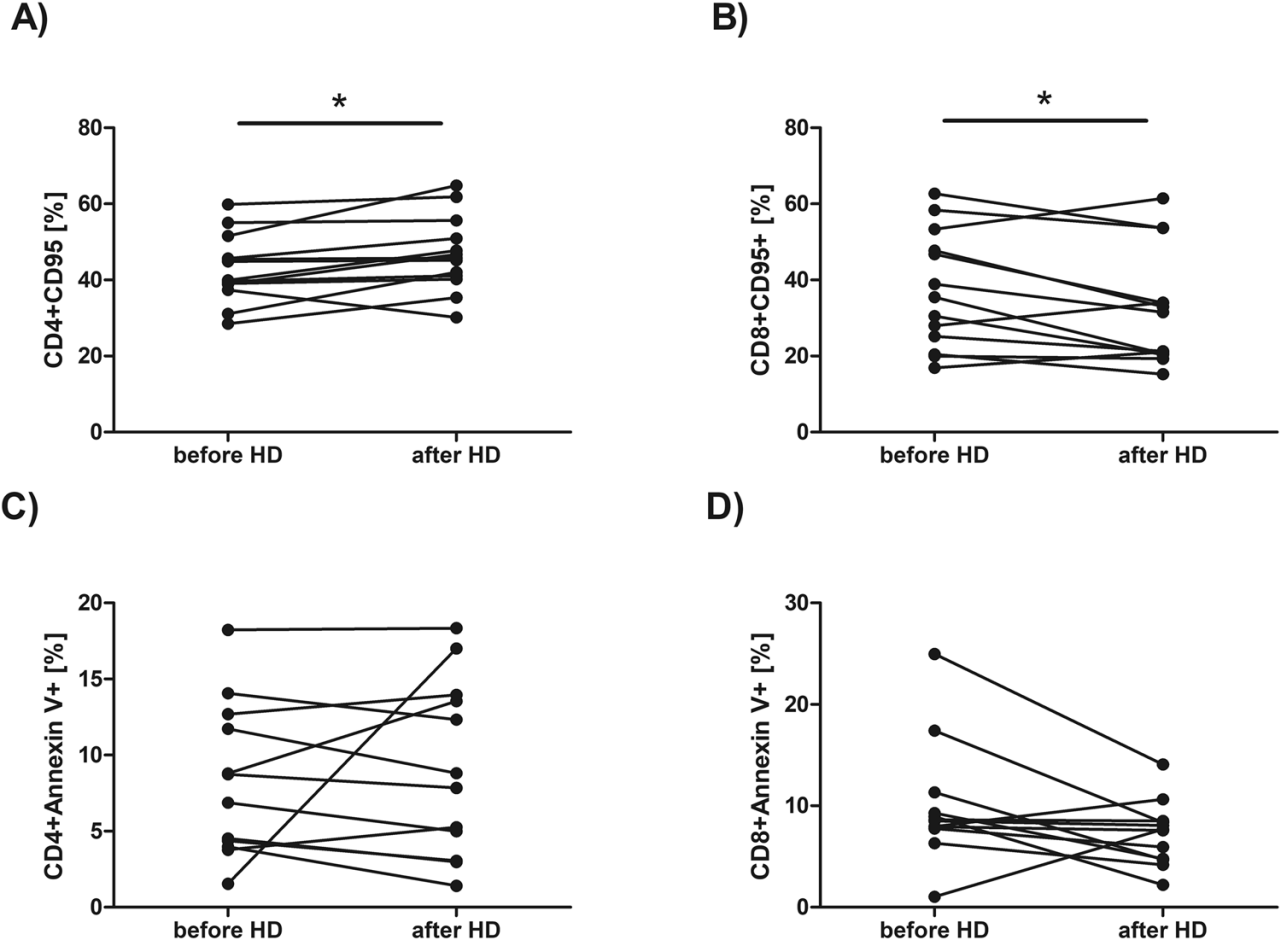
Comparison of the percentage of CD4^+^ and CD8^+^ with the expression of CD95 (**A**,**B**) or annexin V-positive cells (**C**,**D**) in the blood sample taken before and after hemodialysis. Each line shows individual values of cell percentage. Differences statistically significant (p < 0.05) are marked with *, Wilcoxon signed-rank test.

We have found no statistically significant differences in the percentages of CD4^+^ or CD8^+^ cells expressing CD28, or CD25 antigen. The median value of CD4^+^CD28^+^ percentage before HD session was 44,04 *vs* 57,44 after HD while median value of CD8^+^CD28^+^ percentage before HD was 17,25 *vs* 14,35 after HD. The median value of CD4^+^CD25^+^ percentage before HD session was 15,09 *vs* 17,57 after HD while median value of CD8^+^CD25^+^ percentage before HD was 2,21 *vs* 2,25 after HD. No significant difference has also been found in the percentage of CD4^+^HLA-DR^+^ (Fig. 2A) or CD4^+^CD69^+^ cells (Fig. 2C). The median value of CD4^+^HLA-DR^+^ percentage before HD session was 4,33 *vs* 3,15 after HD while median value of CD4^+^CD69^+^ percentage before HD was 2,88 *vs* 1,45 after HD. However, the percentages of CD8^+^HLA-DR^+^ (the median value was 4,38 before HD *vs* 2,93 after HD, p = 0,027709, Wilcoxon signed-rank test) and CD8^+^CD69^+^ (the median value was 3,44 before HD *vs* 1,75 after HD, p = 0,001474, Wilcoxon signed-rank test) cells were significantly decreased after HD procedure (Fig. 2B,D, respectively). The percentage of CD4^+^CD95^+^ cells (Fig. 3A) was significantly increased after HD session (the median value was 39,93 before HD *vs* 45,80 after HD, p = 0,010747, Wilcoxon signed-rank test) while the percentage of CD8^+^CD95^+^ cells was significantly decreased after HD (the median value was 35,51 before HD *vs* 31,49 after HD, Fig. 3B, p = 0,046400, Wilcoxon signed-rank test). No significant difference has been found in the percentage of apoptotic (annexin V-positive) CD4^+^ (the median value was 7,81 before HD *vs* 8,33 after HD, Fig. 3C) or CD8^+^ cells HD (the median value was 8,56 before HD *vs* 7,65 after HD, Fig. 3D) after HD procedure. Also, no difference has been found in the percentage of necrotic (7-AAD-positive) cells after a single HD session (the median value was 3,69 before HD *vs* 3,30 after HD).

**Figure 4:**
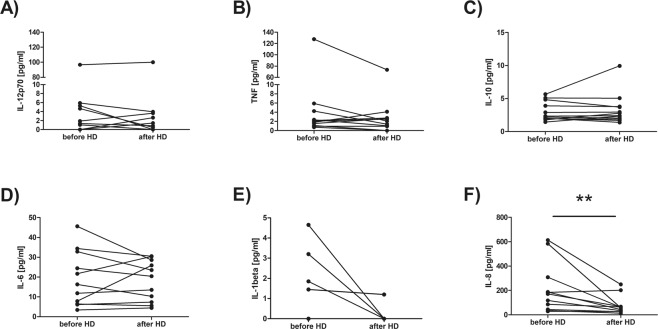
Comparison of IL12p70 (**A**), TNF (**B**), IL-10 (**C**), IL-6 (**D**), IL-1β (**E**) and IL-8 (**F**) concentrations in plasma samples taken before and after hemodialysis. Each line shows individual values of cytokine concentration in pg/ml. Differences statistically significant are marked with **(p < 0,01), Wilcoxon signed-rank test.

Meanwhile, from all analyzed in HD patients plasma samples cytokines, the only difference has been found in the concentration of IL-8 (Fig. 4F), which was significantly decreased after HD procedure (the median value was 171,2 before HD *vs* 57,35 after HD, p = 0,007646, Wilcoxon signed-rank test).

### Comparison of proliferation parameters of in vitro activated T lymphocytes

**Figure 5:**
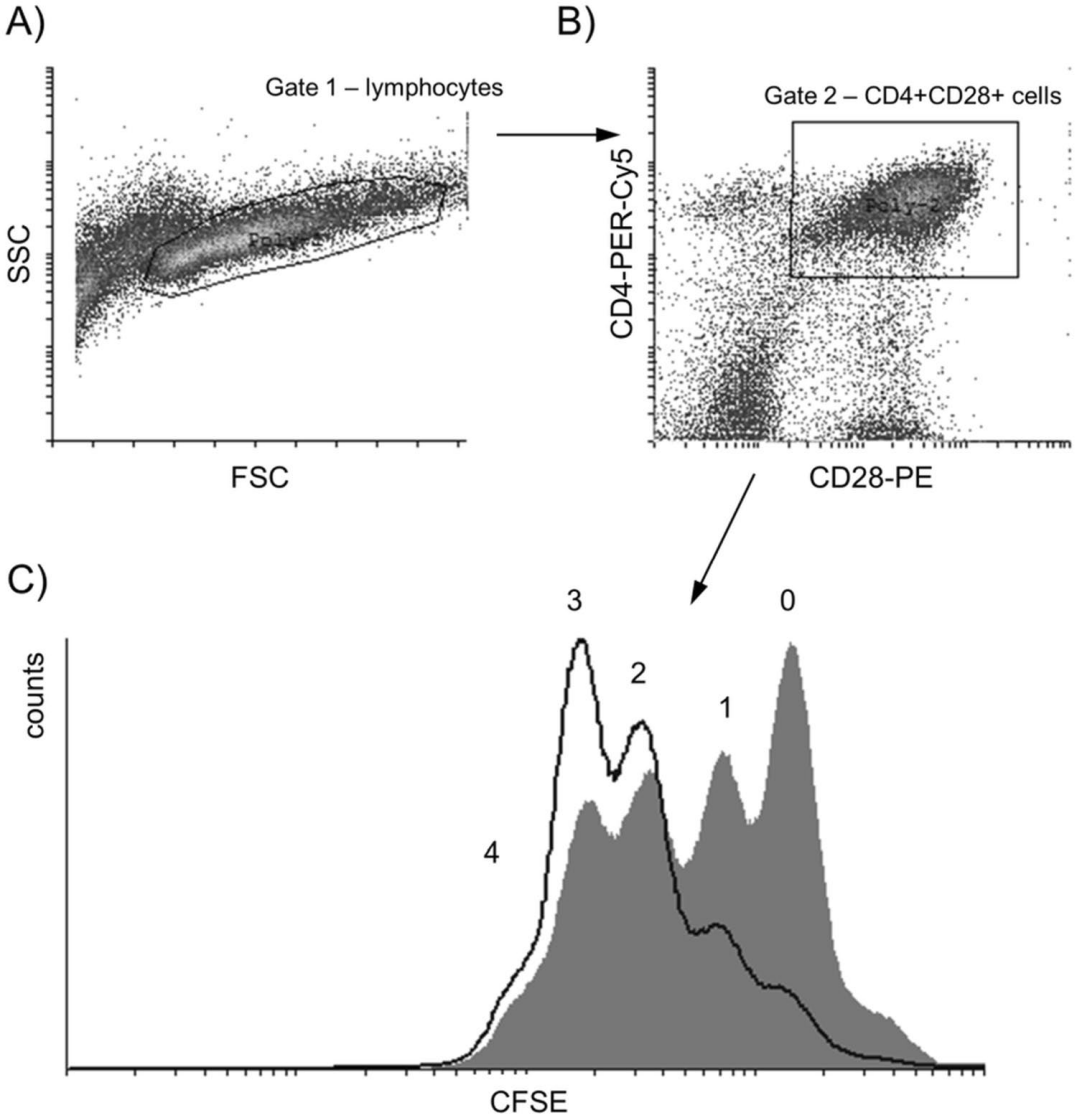
The gating strategy. Proliferating lymphocytes were selected on the basis of forward (FSC) and side scatter (SSC) characteristic (**A**). Next, cells were chosen based on the expression of surface antigens CD4 and CD28 (**B**) and their proliferation was shown as halving the CFSE fluorescence with each division (**C**) − dividing cells obtained before HD are shown as shaded histogram, dividing cells obtained after HD are shown as histogram drawn with a black line, and the numbers of consecutive generations (0–4) are indicated.

In the anti-CD3 stimulated PBMC samples from 7 HD patients before and after single hemodialysis procedure dynamic parameters of proliferation kinetics of CD4^+^ and CD8^+^ T cells were measured with DCT method and analyzed using our own protocol published and validated earlier^11,14^. Proliferating lymphocytes were selected on the basis of forward and side scatter characteristics (Fig. 5A) and then on their positivity for surface antigens (Fig. 5B). A preliminary comparison of the same parameters in HD patients before and immediately after hemodialysis have shown that CD4^+^CD28^+^ cells proliferate more efficiently after HD procedure (Fig. 5C).

**Figure 6:**
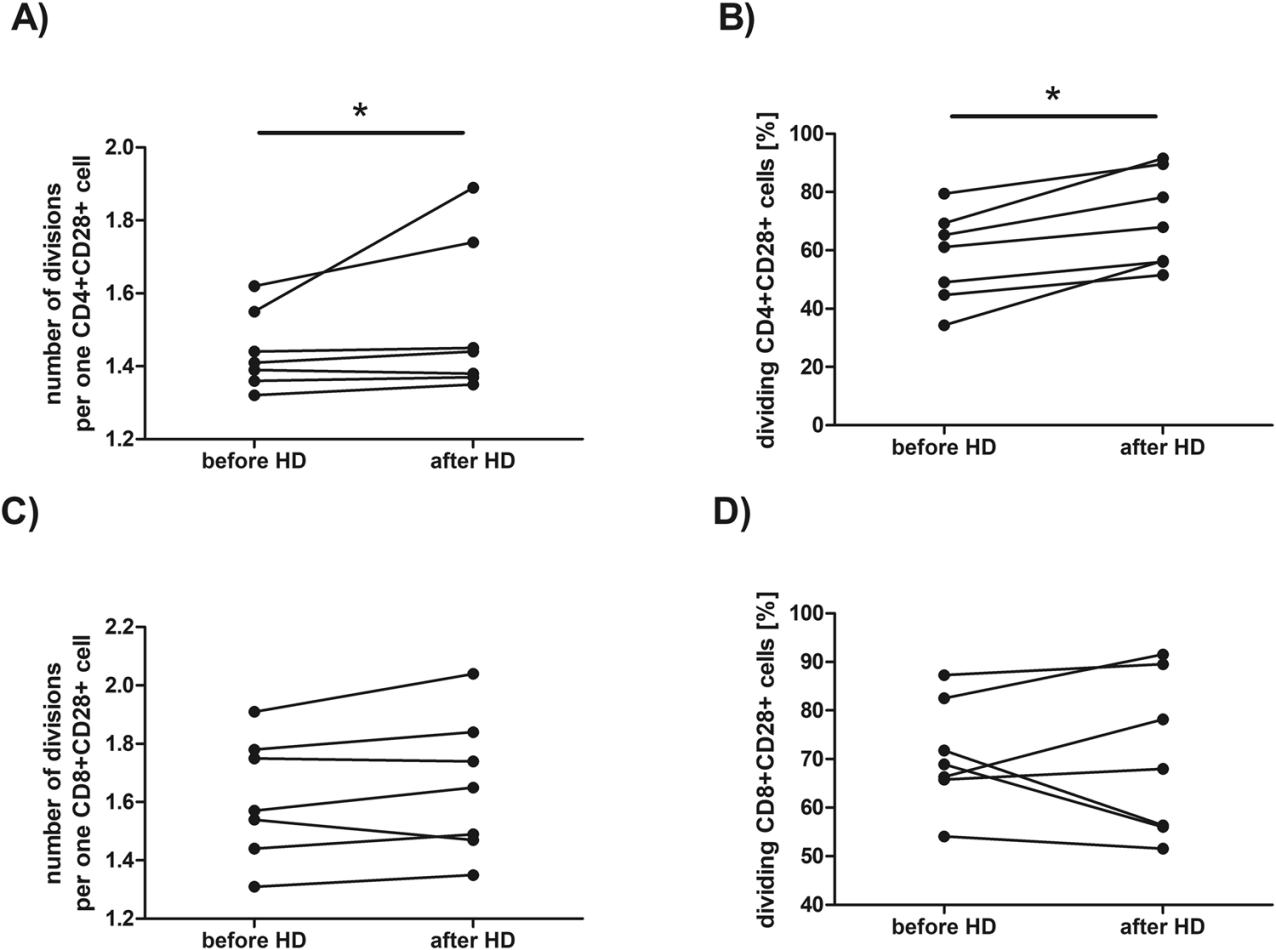
Comparison of the dynamic proliferation parameters of CD4^+^CD28^+^ or CD8^+^CD28^+^ cells after stimulation with immobilized anti-CD3 antibody for 72 hours before and after hemodialysis. Figures (**A**,**C**) present numbers of division per one cell, figures (**B**,**D**) show percentage dividing cells. Each line shows individual values. Differences statistically significant are marked with *(p < 0,05), Wilcoxon signed-rank test.

Figure 6 presents detailed comparison of the selected proliferation parameters of CD4^+^CD28^+^ or CD8^+^CD28^+^ cells after 72 hour of stimulation with anti-CD3 antibody, that is: a number of divisions per one cell (Fig. 6A,C) and percentage of dividing cells (Fig. 6B,D) before and after single HD session. Following hemodialysis CD4^+^CD28^+^ cells (Fig. 6A) of HD patients performed a significantly higher number of cell divisions per one cell (the median value before HD was 1,41 *vs* 1,44 after HD, p = 0,042523, Wilcoxon signed-rank test). The percentage of proliferating CD4^+^CD28^+^ cells (Fig. 6B) was also significantly (the median value before HD was 61,70 *vs* 67,97 after HD, p = 0,017961, Wilcoxon signed-rank test) increased after hemodialysis procedure. Meanwhile, there was no difference in number of cell divisions per one cell in CD8^+^CD28^+^ population (the median value before HD was 1,57 *vs* 1,65 after HD, Fig. 6C) or the percentage of proliferating CD8^+^CD28^+^ cells (the median value before HD was 68,92 *vs* 67,97 after HD, Fig. 6D). However, after 120 hours of stimulation no difference has been found in these proliferation parameters.

## Discussion

Majority of scientific articles describing the abnormal function of the immune cells, especially T lymphocytes, in ESRD is based on the results obtained predominately in HD patients. However, most of our present knowledge derives from the studies carried out many years ago when patients were dialyzed using cellulosic membrane dialyzers (usually cuprophan or hemofan), which are characterized by a lower biocompatibility compared to currently used synthetic alternatives. Also, many studies do not even provide any information on the type of dialysis membranes used during hemodialysis procedures. Furthermore, in most studies the blood of HD patients is usually taken before starting HD session, what only allows to evaluate function of T lymphocytes that has been exposed to uremic toxins for some time (usually about 48 hours). Consequently, we do not exactly know how the procedure itself influences T lymphocyte function.

Along with the development of hemodialysis procedure, cellulosic dialysis membranes have been replaced with synthetic ones, which are believed to be biocompatible. However, our previous study demonstrated that CD4^+^ T lymphocytes of ESRD patients treated with continues hemodialysis have significant disturbances in surface phenotype and reduced proliferation capacity compared with predialysis patients even though synthetic membranes are used^11^. We thought then that these changes could be a result of kidney disease itself or repeated hemodialysis procedure. However, as many other authors, we studied these parameters in the morning before HD, never after the procedure. Therefore, in presented study we decided to analyze if there any changes in the main subpopulations of T lymphocytes in blood taken from the same individual before and immediately after single HD session. First of all, we have found that the percentage of CD3^+^CD8^+^ cells decreases after HD session, which contributed to growth of CD4^+^/CD8^+^ ratio. Interestingly, no difference has been found in the percentage of total T cells (CD3^+^ cells) after HD. Also, we have found no differences in the percentage of CD3^+^CD4^+^ cells. The percentages of CD8^+^HLA-DR^+^, CD8^+^CD69^+^ or CD8^+^CD95^+^ subpopulations also were significantly decreased after HD procedure, probably because the overall percentage of CD8 cells was lower. Since Borges *et al*.^16^ suggested that T cell lymphopenia, which is observed after hemodialysis, could be related to enhanced cell apoptosis, we also analyzed percentages of apoptotic as well as necrotic cells but we found no difference in the percentage of apoptotic CD4^+^ or CD8^+^ cells after HD. Although we did not find necrotic cells in the blood samples obtained after HD, we cannot exclude the possibility that CD8^+^ cells undergo necrosis during the procedure and their cell fragments are removed from patient’s blood by the complement system^17^. It has been demonstrated that presently used polysulfone membranes are capable of lectin pathway activation^18^. Mares *et al*. showed that plasma ficolin-2 (produced by the liver lectin-type pattern recognition receptors) levels are decreased during one HD session, probably because it is adsorbated to the membrane^18^. On the other hand, adsorption of properdin to the dialyzer could be responsible by alternate pathway activation^19^. The complement system activation during HD would result in the recruitment and activation of leukocytes and finally in the release of pro-inflammatory cytokines and chemokines, such as IL-1β, IL-6, IL-8 or TNF-α. An important effect of the complement system stimulation would be the activation of T lymphocytes, especially CD4^+^ cells, which express complement receptors (C3aR, C3bR, C5aR) on their surface^20^. This would also explain increase percentage of CD4^+^CD95^+^ cells; CD95 antigen is a well-known activation antigen of T cells. On the other hand, recently studies of Wang and colleagues demonstrated that complement system can inhibit activity of CD8^+^ cells^21^.

Although we have found no differences in the percentage of CD25^+^ or CD69^+^, which also are well known activation markers of lymphocytes, we cannot exclude possibility that a single hemodialysis procedure could prime the activation of peripheral blood mononuclear cells. Donati *et al*. using an *in vitro* dialysis model with cellulosic or synthetic dialyzers showed that, even though a single session of hemodialysis doesn’t change the percentage of CD25^+^ cells, it increases the expression of mRNA encoding the IL-2R in blood mononuclear cells, which would support the thesis of the premature T cell activation during HD procedure^22^. Expression of the CD25 antigen, which is α chain of IL-2R, is tightly regulated at the transcriptional level and it requires at least 24 hours before it appears at the lymphocyte’s surface^23^. Similar situation concerns CD69 expression, which also requires couple of hours before it becomes detectable on a cell^24^. Since hemodialysis procedure usually lasts about 4 hours, this would explain why we were not able to notice statistically significant differences in the percentage of cells expressing these antigens immediately after HD.

Donati *et al*. in their study also analyzed proliferation capacity of PBMCs taken in the middle (at 20 and 120 minutes) of dialysis session but found no significant difference in the percentage of proliferating cells. We did a similar experiment but we stimulated PBMCs isolated from blood which was obtained after patients finished HD. To our surprise, a preliminary comparison of the same parameters before and after the procedure have shown that CD4^+^ cells seem to proliferate more efficiently by performing more cell divisions and producing more effector cells after HD, which would support the thesis that T cells are indeed prematurely activated during HD session. At the same time, proliferation capacity of CD8^+^ cells wasn’t affected by the procedure. This may be due to the fact that CD8^+^ T cells, compared with CD4^+^ cells, undergo extensive proliferation in response to stimulation with mitogens^25^ or, as mentioned above, activation of CD4^+^ cells is the result of complement activation during hemodialysis^20^. Studies have shown that, in addition to TCR activation and co-stimulation through CD28 antigen, CD4^+^ cells can be activated by C3a and C3b fragments of C3 and further differentiate towards the Th1 responses^26^. This would explain why there is an increase in IFN-gamma-producing Th1 lymphocytes in hemodialysis patients^27^ and why anti-CD3 stimulated T cells of HD patients produce IFN-γ in cell culture^28^. We cannot rule out the influence of other cells, which can also be activated during dialysis. Recent study of Liakapoulos *et al*. demonstrated significant alterations of monocyte subpopulations in hemodialysis patients, including increase of expression of scavenger receptors compared with healthy people as well as up-regulation of TLRs directly after HD procedure^29^.

Our preliminary study supports the thesis that a single hemodialysis can affect proliferation parameters of T lymphocytes, mainly of CD4^+^ cells. Activation of elements of innate immunity during the procedure can lead to pre-activation of T lymphocytes, which we observe as a temporary improvement of their proliferative capacity *in vitro* directly after finished HD session. Repeating this process eventually would lead to exhaustion of the lymphocyte activation capacity. This process is called a stress-induced premature senescence (SIPS), which involves changes in cells’ function and morphology and can be detected, among others, by telomeres shortening. This phenomenon has already been reported in HD patients − in 2005 Jimenez *et al*. demonstrated that mean telomere length is significantly decreased in mononuclear cells (both monocytes and lymphocytes) from HD patients^30^. Moreover, CD4^+^ T lymphocytes of HD patients regularly undergoing HD sessions present lower expression of crucial antigens like CD28, CD69 or CD25 expression^10,11^, which is another sign of their proliferative senescence^31^.

We also determined the level of different cytokines involved in inflammatory responses, that are IL12p70, TNF, IL-10, IL-6, IL-1β, IL-8, in patients’ plasma samples, but we only found decrease in the IL-8 concentration after HD session. We also saw a downward trend in the level of IL-1β after hemodialysis but most patients had undetectable IL-1β before HD. Authors do not agree on what is happening with the cytokine levels during hemodialysis^5,32,33^, which may be related to the variety of dialysis membranes that are used. In our opinion, observed decrease of IL-8 and IL-1β concentrations after single HD session indicates that hemofiltration carried out using polyethersulfone dialyzers increases their clearance, which is definitely beneficial for a patient. However, the lack of decrease in the level of other pro-inflammatory cytokines after HD may be related to excessive monocyte activity during the procedure and would further support thesis of pre-activation of T lymphocytes.

In our opinion, presented study shows that hemodialysis is a very important factor contributing to the state of immune deficiency observed in patients with ESRD. Even tough presently used dialysis membranes seem to be not only very efficient in removing pro-inflammatory cytokines but also more biocompatible, we cannot rule out that CD4^+^ T lymphocytes undergo premature activation during hemodialysis due to excessive activity of elements of innate immune response (e.g. complement system, monocytes) during the procedure. Repeated HD sessions in our opinion eventually lead to exhaustion of T lymphocytes − a process which is well described in human immunology and observed in many other chronic diseases.

## Materials and Methods

### Patients

The study groups consisted of 14 HD patients of mean age of 70,36 ± 12,45 years (10 men and 4 women). The mean time of ESRD duration was 9,46 ± 7,30 months. All patients had glomerular filtration rate (GFR) below 15 min/ml/1.73 m^2^ and underwent 5 h sessions of hemodialysis three times a week using low-flux NIPRO PES 150DL, 170DL, 210DL or high-flux ELISIO 15 H and 17 H dialyzers. 7 patients underwent HD in the morning, 5 ‒ in the afternoon and 2 ‒ in the evening. Patients had regular dialysis adequacy assessment by the measurement of urea clearance using equation Kt/V (K ‒ urea clearance, t ‒ time on dialysis, V ‒ volume of distribution); mean Kt/V was 1,50 ± 0,26. In 4 patients the primary cause of chronic kidney disease was glomerulonephritis, in 3 ‒ diabetic nephropathy, in 2 ‒ adult polycystic kidney disease, in 4 ‒ ischemic nephropathy. In one person the primary cause of CKD was unknown. None of the patients suffered from any infection, inflammation, symptoms of malnutrition, malignancy or blood loss during the study.

All participants were informed about the purpose of the tests and gave their written informed consent; the study has been approved by the Bioethical Committee for Scientific Research at the Medical University of Gdansk. All methods were performed in accordance with the relevant guidelines and regulations.

20 ml of venous peripheral blood was collected from each HD patient immediately before and after HD session in tubes containing EDTA as the anti-coagulant after overnight fasting for the cytometric analysis of lymphocyte subpopulations and peripheral blood mononuclear cells (PBMCs) stimulation. 5 ml of blood was collected into anticoagulant-free tubes in order to collect serum for the assessment of cytokine concentrations. Serum samples were stored at −80 °C.

### Determination of lymphocyte subpopulations *ex vivo*

Samples of 50 μl per tube blood were transferred for staining with monoclonal antibodies and red blood cells (RBCs) lysis. RBCs were lysed with buffer containing 0,8% NH_4_Cl and 0,1% KHCO_3_. Cells were then washed with PBS (phosphate buffered saline) buffer and stained with: FITC-conjugated anti-CD3, R-phycoerythrin-Cy5 (RPE-Cy5)-conjugated anti-CD4 or anti-CD8 (Dako, Denmark) and phycoerythrin (PE)-conjugated anti-CD28, anti-CD25, anti-CD69, anti-HLA-DR or anti-CD95 (BD Pharmingen, USA) for 30 minutes at 4 °C in the dark. After this time cells were washed with PBS, stained with annexin V and 7-amino-actinomycin D (7-AAD) (BD Pharmingen, USA) and finally suspended in 200 µl of suitable buffer for flow cytometric analysis using FACScan instrument (Becton Dickinson, USA).

### PBMC *in vitro* stimulation and dividing cell tracking

PBMCs were isolated by centrifugation on Histopaque™ gradient (Sigma Chemical Co., USA) from venous peripheral blood collected in tubes containing EDTA as the anti-coagulant. Cells were then stained with 3 µM carboxyfluorescein diacetate succinimidyl ester (CFDA-SE, Sigma Chemical Co., USA), stimulated with 250 ng per 2 million cells of immobilized (tissue-culture plate-bound) anti-CD3 antibody (BD-Pharmigen, USA) and incubated for up to 5 days at 37 °C, 5% CO_2_ as previously described^11^.

Stimulated cells were collected after 72 and 120 hours, stained with following antibodies: RPE-Cy5-conjugated anti-CD4 or anti-CD8 and PE-conjugated anti-CD28 (BD Pharmingen, USA), and analyzed with flow cytometry using FACScan instrument.

### Cytokine measurement in plasma samples

BD™ Cytometric Bead Array (CBA) Human Inflammatory Cytokines Kit (BD Biosciences, USA) was used according to manufacturer’s protocol to determine the level of different cytokines: IL12p70, TNF, IL-10, IL-6, IL-1β, IL-8 in the plasma samples of HD patients before and after HD session. Quantitative cytometric fluorescence analysis was performed with FACScan cytometer (Becton Dickinson, USA) in the Department of Pathophysiology, Medical University of Gdańsk. Cytokine concentrations were analyzed with the use of Becton Dickinson software.

### Analysis and Statistics

The gating strategy was as following: cells belonging to the population of interests were selected on the basis of forward and side scatter characteristics and then on their positivity for surface antigens. Twenty thousand events corresponding to the light scatter characteristics of viable lymphocytes were acquired from each sample in order to analyze lymphocyte subpopulations *ex vivo*, thirty thousand events - in order to perform the dividing cell tracking (DCT) method.

DCT method is one of the cell-tracking assays using green fluorescent protein labelling dye, CFDA-SE (carboxyfluorescein diacetate succinimidyl ester) which changes into CFSE (carboxyfluorescein succinimidyl ester) inside the cells. At the time of cell division, the CFSE is distributed in half to the daughter cells, resulting in a 50% reduction in fluorescence, as seen in the cytometer, which allows examining the cell cycle kinetics of T lymphocytes^34^. Obtained raw FACS data were then analysed with Cyflogic software (©Perttu Terho & ©CyFlo Ltd. Turku, Finland). Each peak presented in the histogram (Fig. 5C) corresponds to consecutive generations of dividing (CD4^+^CD28^+^ or CD8^+^CD28^+^) cells. Percentage of dividing cells was calculated by percentage assessment of cells in subsequent generations in relation to all living cells in the population of interest. Calculation of number of divisions per one cell was performed with our own program (Progeny®, by Witkowski J.M.), which uses mathematical equations described in article written by Witkowski^35^.

Statistical analysis was done using the Statistica program, version 10 (StatSoft Inc., Tulsa, Oklahoma, USA). To compare data obtained before and after HD, we used the Wilcoxon signed-rank test with the level of significance p < 0.05. Figures were prepared with GraphPad Prism 5 (GraphPad Software Inc., USA).
